# A Magnetic Wormhole

**DOI:** 10.1038/srep12488

**Published:** 2015-08-20

**Authors:** Jordi Prat-Camps, Carles Navau, Alvaro Sanchez

**Affiliations:** 1 Departament de Física, Universitat Autònoma de Barcelona, 08193 Bellaterra, Barcelona, Catalonia, Spain

## Abstract

Wormholes are fascinating cosmological objects that can connect two distant regions of the universe. Because of their intriguing nature, constructing a wormhole in a lab seems a formidable task. A theoretical proposal by Greenleaf *et al.* presented a strategy to build a wormhole for electromagnetic waves. Based on metamaterials, it could allow electromagnetic wave propagation between two points in space through an invisible tunnel. However, an actual realization has not been possible until now. Here we construct and experimentally demonstrate a magnetostatic wormhole. Using magnetic metamaterials and metasurfaces, our wormhole transfers the magnetic field from one point in space to another through a path that is magnetically undetectable. We experimentally show that the magnetic field from a source at one end of the wormhole appears at the other end as an isolated magnetic monopolar field, creating the illusion of a magnetic field propagating through a tunnel outside the 3D space. Practical applications of the results can be envisaged, including medical techniques based on magnetism.

Constructing an artificial gravitational wormhole connecting two distant regions in the universe is an apparently unrealizable challenge. Large amounts of negative gravitational energy would be required, which makes impossible its realization with present technology^1^,^2^. Greenleaf *et al.* presented a theoretical proposal for designing a wormhole for electromagnetic waves^3^. Such an object could allow electromagnetic wave propagation between two points in space through an invisible tunnel. It would use bulk metamaterials with complicated permeability and permittivity parameters. Actually, metamaterials have enabled unprecedented control of electromagnetic waves^4^,^5^,^6^,^7^, including some realizations mimicking ‘celestial’ objects^8^,^9^,^10^. The design of Greenleaf *et al.*^3^ would effectively change the topology of space, since it would make electromagnetic waves propagate as if they were propagating in 3D-space with an invisible tunnel connecting two distant regions. An alternative theoretical proposal, based on plasmonics, was presented in Ref. 11, not for a full 3D electromagnetic wormhole, but for a 2D analog. However, building an actual 3D electromagnetic wormhole following the strategy in Ref. 3 is very difficult in practice. Among other technical difficulties, it requires cloaking a 3D object like the bulk of the wormhole from electromagnetic waves, and this has not been achieved^12^. All attempts to build cloaks for electromagnetic waves based on transformation optics have been made assuming simplifications such as sacrificing directionality^13^, limiting to a 2D case with a given polarization^14^, or assuming a diffusive medium^15^. Scattering cancellation^12^,^16^ is an alternative strategy, but has the extra problem that it is not suited when the object to be cloaked is large compared to wavelength^12^, a necessary condition for making a wormhole of arbitrary length. Therefore, an actual realization of an electromagnetic wormhole has not been possible until now.

In this work we construct an actual 3D wormhole for magnetostatic fields. It will allow the passage of magnetic field between distant regions while the region of propagation remains magnetically invisible. Although inspired by the theoretical proposal of Greenleaf *et al.*^3^, we do not design our wormhole based on transformation optics. Instead, we take advantage of the possibilities that magnetic metamaterials offer for shaping static magnetic fields. Magnetic materials naturally exist with extreme magnetic permeability values ranging from zero - superconductors - to effectively infinity - ferromagnets -, through many intermediate values^17^,^18^. Metamaterials built from different combinations of them have enabled the realization of magnetic cloaks^19^,^20^,^21^ and other devices for shaping magnetic fields^22^,^23^,^24^,^25^.

## Results

**Figure 1:**
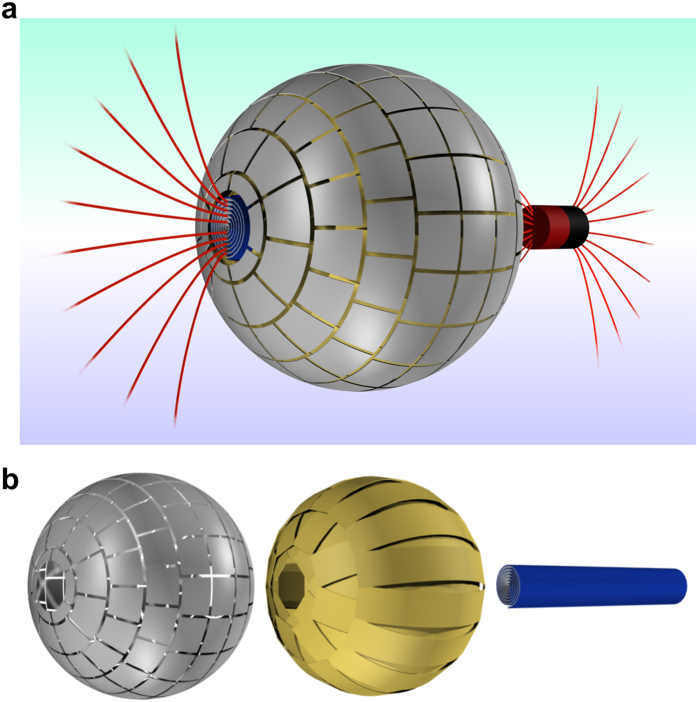
(**a**) The field of a magnetic source (right) is appearing as an isolated magnetic monopole when passing through the magnetostatic wormhole; the whole spherical device is magnetically undetectable. (**b**) The wormhole is composed of (from left to right) an outer spherical ferromagnetic metasurface, a spherical superconducting layer, and an inner spirally wound ferromagnetic sheet.

The magnetostatic wormhole requires constructing a tunnel for magnetic fields acting as if was outside the usual 3D space. The first property to be satisfied is to magnetically decouple a given volume from the surrounding 3D space. The volume enclosed by a superconducting shell has this property^19^; we consider here a spherical superconducting shell (Fig. 1b, center). A second property is that the whole resulting wormhole cannot be magnetically detectable from its exterior. The superconducting spherical shell would distort an applied field, and thus be detectable. In Ref. 21, a cylindrical magnetic cloak was made consisting of a superconducting layer surrounded by a magnetic layer; similar bilayer structures have been recently applied to thermal and diffusive cloaks^15^,^26^,^27^. However, the magnetic bilayer was actually 2D - a long cylinder- and was shown to cloak only uniform applied magnetic fields. Realizing an actual magnetic wormhole requires it to be fully 3D and undetectable also for non-uniform fields. To solve this challenge we demonstrate (see Supplementary Information) that a spherical bilayer composed of a superconducting layer of outer radius *R*_2_ surrounded by a ferromagnetic layer of outer radius *R*_3_ exactly cloaks a uniform applied magnetic field if the permeability of the ferromagnetic layer is


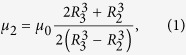


where *μ*_0_ is the vacuum permeability. Interestingly, when the applied field is not uniform such a bilayer also cancels the dipolar term of its magnetic response. Furthermore, all the terms of the magnetic response higher than the dipole can be reduced by making *R*_3_ tend to *R*_2_, that is, by thinning the ferromagnetic layer. This means a very thin ferromagnetic layer with permeability *μ*_2_ surrounding the superconducting shell effectively cancels the global magnetic response. Since the superconducting and ferromagnetic layers will also be magnetically decoupling the region enclosed by them, then the overall effect is changing the topology of space^3^, as if the interior region had been (magnetically) removed out of the existing 3D space (Fig. 1).

A final requirement for the wormhole is that magnetic fields have to propagate through its interior. Magnetic fields naturally decay with distance, typically as a magnetic dipole (field at a distance *d* is decreasing as 1/*d*^3^). In Ref. 25 a general strategy for transferring magnetic field to long distances was developed. There a magnetic hose consisting of a ferromagnetic tube surrounded by a superconductor was employed. Here we use an alternative option, namely a thin ferromagnetic sheet wound into a spiral (Fig. 1b). This scheme was also theoretically shown to yield a good field transfer at a distance^25^; here it is experimentally confirmed.

The parts composing the wormhole are shown in Fig. 1b (see also Supplementary Information). The core of the device is the magnetic hose made of a foil of high-permeability mu-metal, folded into a spiral. This is surrounded by the spherical superconducting layer, made of high-temperature superconducting strips glued to a spherical former to provide a tessellation of the sphere. On top of it, there is an outer ferromagnetic layer, ideally made of a homogenous material with permeability *μ*_2_ given by Eq. (1), and very small thickness. Such effective permeability is achieved by a strategy reminiscent of the metasurfaces used for light manipulation^28^. An array of high-permeability mu-metal plates is specially arranged as to provide the required magnetic response, following an optimization process based on a 3D-numerical simulation of the whole device (see Supplementary Information).

**Figure 2:**
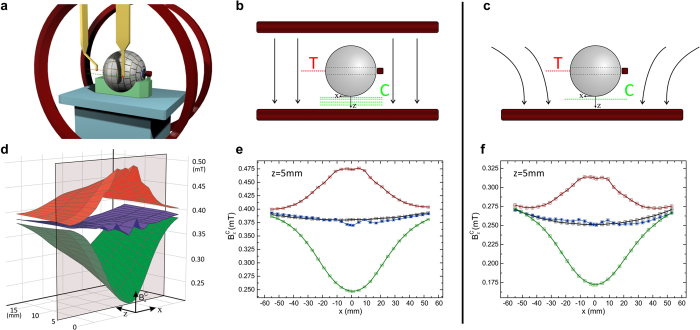
(**a**) 3D scheme of the experimental setup. (**b**) A detailed description of the central plane, including the lines at which probes T (red) and C (green) measure the transferred and cloaked (or distorted) fields, respectively. The uniform applied field is created by the two Helmholtz coils. (**d**) In this case, the *z*-component of magnetic field is measured by probe C as a function *x* and for different distances, *z*, to the wormhole. (**e**) Measurements at *z *= 5 are shown in detail. (**c**) Analogous measurements are done for a non-uniform applied field, created by exciting only one of the coils, and results are shown in (**f**). Red lines are for only the ferromagnetic layer, green for only the superconducting one and blue for the complete device. Black lines represent the measured applied field for each case.

The experimental setup to demonstrate the wormhole properties (see Fig. 2 and Supplementary Information) uses a pair of Helmholtz coils of radius *R* separated a distance *R*. They provide a uniform magnetic field in the central zone. There sits the wormhole, oriented with its two ends perpendicular to the applied field. A small coil at one end of the wormhole is fed with a dc current to supply the field to be transferred through it. Two Hall probes are used for the measurements. Probe T, placed at the opposite exit of the wormhole measures the transferred magnetic field. Probe C scans the magnetic field in lines (green lines in Fig. 2b) close to the surface of the wormhole, measuring the distortion of the applied field. Three types of measurements are performed by probe C: (i) only the superconducting layer, without the ferromagnetic outer layer (this measurement requires submerging the superconductor into liquid nitrogen); (ii) only the ferromagnetic layer (actually measuring the whole device at room temperature, with the superconductor deactivated); and (iii) the full structure, at liquid nitrogen temperature, so that both superconducting and ferromagnetic layers are activated. Ideally, one should observe a clear field distortion of the applied magnetic field in cases (i) and (ii) and no distortion for the fully working wormhole of case (iii). Accurate 3D simulations by finite elements of the whole device, considering details like the ferromagnetic metasurface, validate the design (see Supplementary Information).

We next present the experimental results. Although measured simultaneously, we discuss the transmission and cloaking results separately for clarity. Results of the field distortion for the three cases (i)–(iii) at a distance of 5 mm are shown in Fig. 2e. The complete wormhole of case (iii) shows an excellent cloaking behavior, whereas the superconducting and ferromagnetic parts separately yield clear field distortions. Scans performed at different distances show very little distortion (see Fig. 2d and Supplementary Information). Only at a close distance the effect of the non-uniform ferromagnetic structure can be discerned. We also measure the effect of applying a non-uniform applied field, created by feeding current in only one of the Helmholtz coils (Fig. 2c,f). Even in this case, a very good cancellation of the field distortion is achieved for the full wormhole and not for its components separately. In this way we confirm the magnetic undetectability of the wormhole.

**Figure 3:**
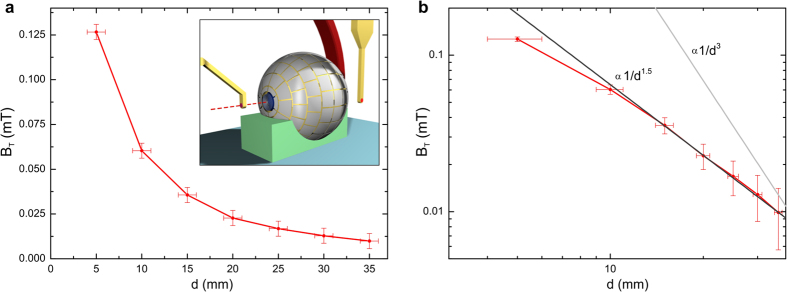
Measurements of the horizontal component of magnetic field measured by probe T as a function of distance in (**a**) linear, and (**b**) double-logarithmic scales. Field shows a dependence with distance *d* roughly as ∼1/*d*^1.5^, very different from a dipolar dependence ∼1/*d*^3^.

Experiments show a clear transmission of magnetic field through the wormhole as well (Fig. 3). The dipolar-like magnetic field created by the feeding coil at one end of the wormhole is transformed at the opposite end into a monopolar-like field. Actually, the spacial dependence of the exiting field tends to ∼1/*d*^1.5^, since close to the opening the field resembles that of a disk of monopoles rather than a single one (∼1/*d*^2^) . These monopolar magnetic fields are an alternative to those obtained by exotic spin ices^29^ and other systems^30^. Our magnetic wormhole thus creates an illusion of a magnetic field coming out of nowhere.

## Discussion

Although we have constructed a spherical wormhole, similar results can be obtained for the shape of an elongated ellipsoid that could extend to long distances in one direction. These ideas may be applied in devices requiring the local application of magnetic fields in a particular magnetic background that should not be distorted. One particularly relevant application along this line could be in magnetic resonance imaging. Using the ideas in this work, one could foresee ways to apply a magnetic field locally to a patient, without distorting the homogenous magnetic field in the region^3^,^31^. They could be useful, for example, in medical operations using simultaneous MRI imaging^3^.

Two final comments on the validity and exactness of our wormhole. First, both ends of the wormhole have been considered only in an approximate way. Because of the finite openings in the spherical shell, the cloaking properties will not be perfect near these regions. The field distortion at the ends could be reduced by refining the design. Second, our results have been experimentally confirmed only for dc fields. However, both ferromagnets and superconductors have been shown to maintain their properties for low frequencies electromagnetic waves in Refs. 32 and 33, so the wormhole could also be effective at low ac frequencies.

To sum up, we have demonstrated that the ideas in Ref. 3 of effectively changing the topology of space can be realized with magnetic fields, not only as an abstract paradigm^11^, but by constructing an actual 3D spatial wormhole and measuring its properties. Our wormhole appears roughly as a sphere in most regions of the electromagnetic spectrum, including visible light. However, with respect to magnetic fields, the object allows the passage of field lines through its interior while being magnetically invisible. The situation is analogous as having the magnetic field propagating through a handlebody attached to the ***R***^3^ space^3^. In this way, the magnetic field of a dipole entering in one end of the wormhole appears as a monopolar-like field at the other end. These ideas can be useful in practical situations where magnetic fields have to be transferred without distorting a given field distribution, as in magnetic resonance imaging.

## Additional Information

**How to cite this article**: Prat-Camps, J. *et al.* A Magnetic Wormhole. *Sci. Rep.* **5**, 12488; doi: 10.1038/srep12488 (2015).

## Supplementary Material

Supplementary Information

## References

[b1] Morris M. S., Thorne K. P. & Yurtsever U. Time Machines, and the Weak Energy Condition. Phys. Rev. Lett. 61, 1446 (1998). 10038800 10.1103/PhysRevLett.61.1446

[b2] Morris M. S. & Thorne K. P. Wormholes in spacetime and their use for interstellar travel: A tool for teaching general relativity. Am. J. Phys. 56, 395 (1998).

[b3] Greenleaf A. Kurylev Y., Lassas M. & Uhlmann G. Electromagnetic Wormholes and Virtual Magnetic Monopoles from Metamaterials. Phys. Rev. Lett. 99, 183901 (2007). 17995408 10.1103/PhysRevLett.99.183901

[b4] Cui T. J., Smith D. R. & Liu R. Metamaterials: Theory, Design and Applications (New York: Springer, 2010).

[b5] Pendry J. B., Schurig D. & Smith D. R. Controlling electromagnetic fields. Science 312, 1780 (2006). 16728597 10.1126/science.1125907

[b6] Chen H., Chan C. T. & Sheng P. Transformation optics and metamaterials. Nature Materials 9, 387 (2010). 20414221 10.1038/nmat2743

[b7] Zheludev N. I. & Kivshar Y. S. From metamaterials to metadevices. Nature Materials 11, 917 (2012). 23089997 10.1038/nmat3431

[b8] Genov D. A., Zhang S. & Zhang X. Mimicking celestial mechanics in metamaterials. Nat. Phys. 5, 687 (2009).

[b9] Narimanov E. E. & Kildishev A. V. Optical black hole: Broadband omnidirectional light absorber. Appl. Phys. Lett. 95, 041106 (2009).

[b10] Cheng Q., Cui T. J., Jiang X. X., & Cai B. G. An omnidirectional electromagnetic absorber made of metamaterials. New Journal of Physics 12, 063006 (2010).

[b11] Kadic M., Dupont G., Enoch S. & Guenneau S. Invisible waveguides on metal plates for plasmonic analogs of electromagnetic wormholes Phys., Rev. A 90, 043812 (2014).

[b12] Fleury R. & Alù A. Cloaking and Invisibility: A Review. Forum for Electromagnetic Research Methods and Application Technologies (FERMAT) 1, 9 (2014).

[b13] Li J. & Pendry J. B. Hiding under the Carpet: A New Strategy for Cloaking. Phys. Rev. Lett. 101, 203901 (2008). 19113341 10.1103/PhysRevLett.101.203901

[b14] Schurig D. *et al.* Metamaterial Electromagnetic Cloak at Microwave Frequencies. Science 314, 977 (2006). 17053110 10.1126/science.1133628

[b15] Schittny R., Kadic M., Buckmann T. & Wegener M. Invisibility cloaking in a diffusive light scattering medium. Science 345, 427 (2014). 24903561 10.1126/science.1254524

[b16] Chen P.-Y., Soric J. & Alù A. Invisibility and Cloaking Based on Scattering Cancellation. Advanced Materials 24, OP281 (2012). 23080411 10.1002/adma.201202624

[b17] Wood B. & Pendry J. B. Metamaterials at zero frequency. J. Phys. Condens. Matter 19, 076208 (2007). 22251595 10.1088/0953-8984/19/7/076208

[b18] Magnus F. *et al.* A d.c. magnetic metamaterial. Nat. Mater. 7, 295 (2008). 18297077 10.1038/nmat2126

[b19] Sanchez A., Navau C., Prat-Camps J. & Chen D.-X. Antimagnets: controlling magnetic fields with superconductorâ€“metamaterial hybrids. New J. Phys. 13, 093034 (2011).

[b20] Narayana S. & Sato Y. D. C. Magnetic Cloak. Advanced Materials 24, 71 (2012). 22114004 10.1002/adma.201104012

[b21] Gomory F. *et al.* Experimental realization of a magnetic cloak. Science 335, 1466 (2012). 22442477 10.1126/science.1218316

[b22] Navau C., Prat-Camps J. & Sanchez A. Magnetic Energy Harvesting and Concentration at a Distance by Transformation Optics. Phys. Rev. Lett. 109, 263903 (2012). 23368564 10.1103/PhysRevLett.109.263903

[b23] Sun F. & He S. Static magnetic field concentration and enhancement using magnetic materials with positive permeability. Progress In Electromagnetics Research 142, 579 (2014).

[b24] Jung P., Ustinov A. V. & Anlage S. M. Progress in superconducting metamaterials. Supercond. Sci. Technol. 27, 073001 (2014).

[b25] Navau C., Prat-Camps J., Romero-Isart O., Cirac J. I. & Sanchez A. Long-Distance Transfer and Routing of Static Magnetic Fields. Phys. Rev. Lett. 112, 253901 (2014). 25014816 10.1103/PhysRevLett.112.253901

[b26] Han T. C. *et al.*. Experimental Demonstration of a Bilayer Thermal Cloak. Phys. Rev. Lett. 112, 054302 (2014). 24580600 10.1103/PhysRevLett.112.054302

[b27] Xu H., Shi H., Gao F., Sun H. & Zhang B. Ultrathin Three-Dimensional Thermal Cloak. Phys. Rev. Lett. 112, 054301 (2014). 24580599 10.1103/PhysRevLett.112.054301

[b28] Yu Y. N. & Capasso F. Flat optics with designer metasurfaces. Nat. Mat. 13, 139 (2008). 10.1038/nmat3839 24452357

[b29] Castelnovo C., Moessner R. & Sondhi S. L. Magnetic monopoles in spin ice. Nature 451, 42 (2008). 18172493 10.1038/nature06433

[b30] Ray M. W., Ruokokoski E., Kandel S., Mottonen M. & Hall D. S. Observation of Dirac monopoles in a synthetic magnetic field. Nature 505, 657 (2014). 24476889 10.1038/nature12954

[b31] Anlage S. M. Magnetic Hose Keeps Fields from Spreading. Physics 7, 67 (2014).

[b32] Souc J. *et al.* A quasistatic magnetic cloak New Journal of Physics 15, 053019 (2013).

[b33] Prat-Camps J., Navau C. & Sanchez A. Experimental realization of magnetic energy concentration and transmission at a distance by metamaterials Appl. Phys. Lett. 105, 234101 (2014).

