# Supplementary material for: A Magnetic Wormhole
Source: Sci Rep. 2015 Aug 20;5:12488. doi: 10.1038/srep12488 (PMC4542659; doi:10.1038/srep12488)
Supplement: Supplementary Information [file srep12488-s1.pdf]

Supplementary Information for "A Magnetic Wormhole"

Jordi Prat-Camps, Carles Navau, and Alvaro Sanchez
Departament de Física, Universitat Autònoma de Barcelona,
08193 Bellaterra, Barcelona, Catalonia, Spain

I. METHODS

Wormhole construction

The magnetic hose at the core of the wormhole was made of a mu-metal foil 0.2mm thick, folded into a spiral. A cardboard sheet was used to separate the different turns of the spiral. The final hose had a length of 87mm, an exterior diameter of 12mm and had 8 turns of ferromagnetic foil (see Fig. 1B).

The spherical cloaking shell was composed of an inner superconducting (SC) shell surrounded by an external ferromagnetic (FM) metasurface. The superconducting shell was made of several pieces of type-II SC strip 12mm width (SuperPower SF12050) wrapped around a plastic former 3D-printed in PLA thermoplastic (Fig. S1**d**). The former had an external diameter of 79mm and had 8 openings to allow the free circulation of liquid nitrogen. It also had two traversing holes of radius 8mm. On the external surface of this former 8 pieces of SC strip were glued following the meridian lines from one traversing hole to the other, keeping the same spacing between the strips. Then, 8 shorter pieces of strip were placed between the previous ones, partially covering the space between them. These pieces were fixed on top of the previous using small welding points. Finally, 16 shorter pieces of strip were placed to cover the remaining gaps, and were fixed using small welding points. Some pieces of adhesive tape where put to reinforce the fixing points near the two traversing holes (see Fig.1B and Fig. S1**a**).

The external FM shell was made of several pieces of mu-metal foil 0.2mm thick. The distribution and size of the pieces was optimized as explained in Section III of the Supplementary Information. A plastic support with the optimized shape was specially designed and 3D-printed in PLA thermoplastic (see Fig. S1**e**), containing the lodgings for the pieces. We used 155 pieces with 6 different shapes, which where glued in the corresponding lodgings (placing them at a radial distance of 43mm to the center, see Fig. S1**b**). Finally, some pieces of adhesive tape were put covering all the FM pieces.

A virtual image of the complete wormhole is shown in Fig. S1**f**, where the cut allows to see the different parts, including the plastic supports. A real picture of the wormhole is shown in Fig. S1**c**.

Experimental setup

The applied field was created by a pair of Helmholtz coils with a radius of 150mm separated a distance of 150mm. The wormhole was placed approximately at its center, inside a vessel made of polystyrene foam. A lower support for the wormhole was designed and 3D-printed in PLA, keeping it fixed and aligned (see gray lower part and red centering bars in Fig. S1**c**). This support also hold the small coil that created the transmitted field. This coil had and approximate radius of 5mm and length of 10mm, it was aligned with the hose and placed leaving a gap of 2mm between the coil and the wormhole's end.

Measurements where done using two different Hall probes. Probe C measured the distortion created by the device (as a function of the x position) and for different distances from the surface of the device (see Fig. 2B). Probe T measured the field transmitted through the device as a function of the distance from the device's end d ($d = x - x_{\rm surf}$, being $x_{\rm surf}$ the position of device's end).

Measurements

Measurements of the FM shell were performed placing only the FM shell and measuring the field distortion with probe C at room temperature. We also measured the field with probe C when no device and no field was applied (measuring the earth's magnetic field and other ambient fields). These field values were subtracted to obtain the final measurements.

Measurements of the SC shell were performed placing the SC shell and the hose inside and submerging it into liquid nitrogen. Probes were also submerged. Before any field was applied, we measured the field at the exit of the device. Then, the Helmholtz field was applied and the field distortion was measured with probe C. Also the field at the exit was measured. After this the small coil was also fed, and the distortion was measured again with probe C and the transmission with probe T. This same procedure was repeated applying the field of a single coil (deactivating one of the Helmholtz coils). We also measured the field with probes C and T submerged in liquid nitrogen when no device and no field was applied. These fields values were subtracted to obtain the final measurements.

Measurements of the complete wormhole were performed placing the SC and the FM shells and the inner hose, and submerging the whole device and the two probes in liquid nitrogen. We followed the same previous measurement procedure.

Numerical calculations

3D simulations where obtained by the AC/DC module of Comsol Multiphysics software.

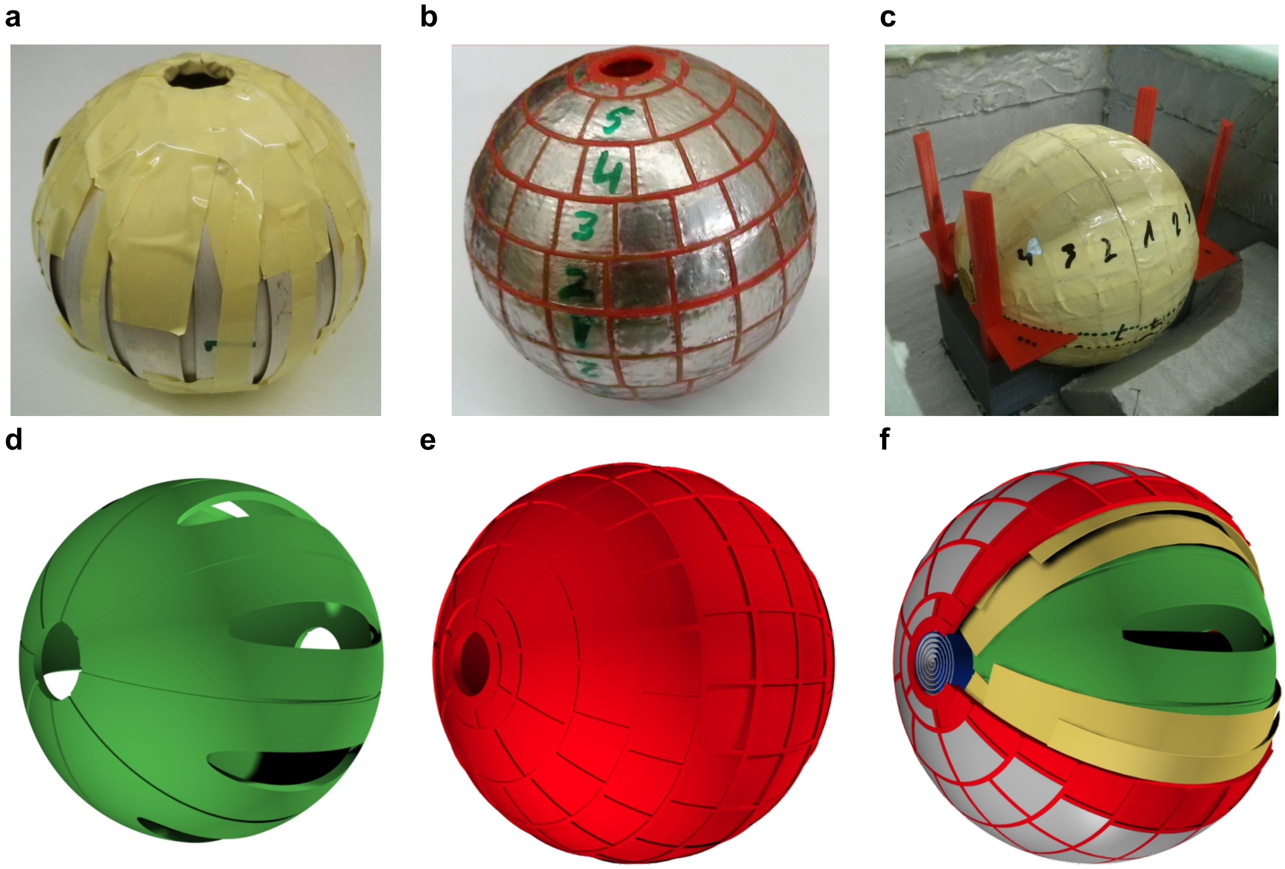

Figure S1: The inner SC spherical shell, **a**, was made of several pieces of type-II SC strip fixed on a plastic former, **d**. The exterior FM metasurface, **b**, was made by fixing 155 pieces of mu-metal on a specially designed plastic former containing the lodgings for the pieces, **e**. Both formers where designed and 3D-printed in PLA thermoplastic. The wormhole, **c**, contained an inner hose traversing the two shells, as can be seen in the cross-section view, **f**.

II. ANALYTICAL SOLUTION OF A SPHERICAL HOMOGENEOUS BILAYER IN THE PRESENCE OF AN EXTERNAL DIPOLAR FIELD

A. The problem and the general solution

Consider a spherical bilayer formed of an interior shell of magnetic material with permeability μ_1 and inner radius R_1 and outer one R_2. This inner layer is wrapped with a second exterior shell with inner radius R_2 and outer one R_3 of magnetic material with permeability μ_2 (both permeabilities are considered homogeneous and isotropic). The interior hole and the exterior region are magnetically empty (μ_0). We use standard spherical coordinates (r, θ, ϕ) centered in the center of the bilayer. When needed, standard cartesian (x, y, z) coordinates will also be used. Hats over the coordinate letter will mean unitary vectors in the corresponding direction (see Fig. S2).

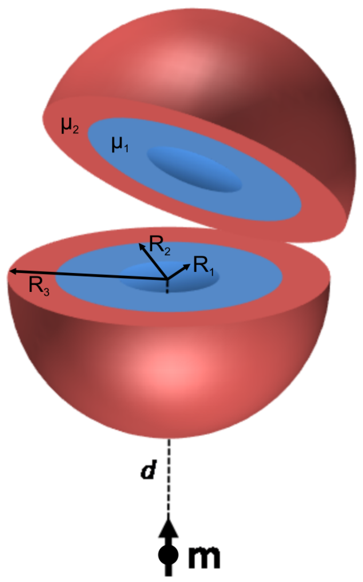

Figure S2: Sketch of the considered bilayer.

Consider an external magnetic dipole located at $-d\hat{z}$ with magnetic moment $\mathbf{m} = m\hat{z}$. The dipole is external to the bilayer, thus $d > R_3$. Since there are no free currents, we can define a magnetic scalar potential $f(r, \theta, \phi)$ such that $\mathbf{H} = -\nabla f$ and $\mathbf{B} = \mu\mathbf{H}$, being μ the corresponding permeability, depending on the region we are considering: H (for 'hole') $r < R_1$, I (for 'interior shell') $R_1 < \rho < R_2$, E (for 'exterior shell') $R_2 < \rho < R_3$, and O (for 'outside') $R_3 < \rho$. The Laplace equation for the scalar potential holds in all the regions of space. Its general solution at the different regions can be written as

$$f_H = \sum_{n=0}^{\infty} A_n r^n P_n(\cos\theta), \tag{S1}$$

$$f_I = \sum_{n=0}^{\infty} \left(B_n r^n + \frac{C_n}{r^{n+1}} \right) P_n(\cos\theta), \tag{S2}$$

$$f_E = \sum_{n=0}^{\infty} \left(E_n r^n + \frac{F_n}{r^{n+1}} \right) P_n(\cos\theta), \tag{S3}$$

$$f_O = f_{dip} + \sum_{n=0}^{\infty} \frac{D_n}{r^{n+1}} P_n(\cos\theta), \tag{S4}$$

where we have already used that the scalar potential should be finite at the origin and should tend to the f_{dip} at infinite. A_n, B_n, C_n, E_n, F_n, and D_n are constants to be determined by the boundary conditions, $P_n()$ are the Legendre polynomials of integer order n ($n \geq 0$), and f_{dip} is the scalar potential of the dipole that can be

expressed as

$$f_{dip} = \frac{m}{4\pi}\frac{d + r\cos\theta}{(d^2 + r^2 + 2dr\cos\theta)^{3/2}},, \tag{S5}$$

or expressed as an expansion of Legendre polynomials as

$$f_{dip} = \begin{cases} \frac{m}{4\pi}\sum_{n=0}^{\infty}(-1)^n \frac{n+1}{d^2}\left(\frac{r}{d}\right)^n P_n(\cos\theta) & \text{if } r < d, \\ \frac{m}{4\pi}\sum_{n=0}^{\infty}(-1)^{n+1}\frac{n}{d^2}\left(\frac{d}{r}\right)^{n+1} P_n(\cos\theta) & \text{if } r > d. \end{cases} \tag{S6}$$

Note that the scalar potential of a dipole not centered at the origin of coordinates, f_{dip}, contains several (actually infinite) terms in the Legendre expansion, with respect to the origin of coordinates.

The standard boundary conditions (continuity of radial component of $\mathbf{B}$ and continuity of tangential component of $\mathbf{H}$) yield in a set of equations for the constants A_n, B_n, C_n, E_n, F_n, and D_n. Because of the orthogonality of the Legendre polynomials and of their derivatives (with cosine argument) with respect to the angle (associated Legendre polynomials) the set of equations can be split in a system of equations for each n. So, for each n, we find a set of six linear algebraic equations for the A_n, B_n, C_n, E_n, F_n, and D_n unknowns. The general solutions of these sets are cumbersome, although straightforward. The terms $n = 0$ are $A_0 = B_0 = E_0 = \frac{m}{4\pi d^2}$, and $C_0 = F_0 = D_0 = 0$, indicating that the term $n = 0$ is a constant that could be removed from the scalar potential terms.

We are here most interested in the distortion of the field outside the bilayer. As can be easily seen from Eq.(S4) this distortion is controlled by the constants D_n. Actually, the 'distortion term' $\sum_{n=0}^{\infty}\frac{D_n}{r^{n+1}}P_n(\cos\theta)$ is a multipolar expansion $n = 1$ being the dipolar term, $n = 2$ the quadrupolar one, and so on. As already mentioned, the constant $D_0 = 0$. We define $\alpha = R_1/R_2$ and $\beta = R_3/R_2$ (note that R_2 will be used as a normalization distance, $\alpha < 1$ and $\beta > 1$) for the following discussions.

B. Multipolar terms in the distortion

From the general expression for D_n, we see that for each n, D_n can be canceled. Thus, for a given n, $D_n = 0$ if

$$\beta = \left(\frac{(n(\mu_0+\mu_2)+\mu_2)\left(\alpha^{2n+1}(\mu_1-\mu_0)(n\mu_1+n\mu_2+\mu_1)+(\mu_1-\mu_2)(-n(\mu_0+\mu_1)-\mu_1)\right)}{(\mu_2-\mu_0)\left(n(n+1)\alpha^{2n+1}(\mu_0-\mu_1)(\mu_1-\mu_2)+(n(\mu_0+\mu_1)+\mu_1)(n(\mu_1+\mu_2)+\mu_2)\right)}\right)^{\frac{1}{2n+1}}. \tag{S7}$$

So, the different terms of the expansion can be separately canceled by conveniently choosing the relation of radius. Note that this relation also depends on the α ratio. In the case $\alpha = 0$ we find

$$\beta = \left(\frac{(\mu_1-\mu_2)(n(\mu_0+\mu_2)+\mu_2)}{(\mu_0-\mu_2)(n(\mu_1+\mu_2)+\mu_2)}\right)^{\frac{1}{2n+1}}. \tag{S8}$$

This last expression has also been found considering the problem of cancellation of the scattering terms when a plane wave of wavelength λ is applied over small ($R_3 \ll \lambda$) spherical layered shells [1, 2]. In this case, because the scatterer is very small as compared with the wavelength, the $n = 1$ term dominates the scattering and the solution reduces to $\sqrt[3]{\frac{(\mu_0+2\mu_2)(\mu_1-\mu_2)}{(\mu_0-\mu_2)(\mu_1+2\mu_2)}}$ (also found in Ref. [3]). This limit would correspond to the case in which the scatterer feels a basically uniform field and radiative terms are not important. In our present case the applied field is not uniform, although static. In consequence, our solution is not based on the small-size approximation, but exact. In general however, not only the dipolar ($n = 1$) term should be considered, but the next multipolar terms can be important.

C. External field distortion when the bilayer has an interior superconducting shell

We now focus on a particular case. Consider here that the interior shell is an ideal superconductor and, thus, consider $\mu_1 = 0$. Actually, $\mu_1 = 0$ yields $A_n = 0$ (for $n > 0$) indicating that no magnetic field is present inside the hole due to the shielding effect of the superconducting layer.

From the general solution and setting $\mu_1 = 0$ we find that

$$D_n = \frac{m}{4\pi} \frac{(-1)^n n R_3^{1+2n}[(1+n)R_3^{1+2n}(\mu_0 - \mu_2) + R_2^{1+2n}(\mu_2 + n(\mu_0 + \mu_2))]}{d^{n+2}[nR_2^{1+2n}(\mu_0 - \mu_2) + R_3^{1+2n}(\mu_0 + n(\mu_0 + \mu_2))]}. \tag{S9}$$

As said, there is no way of canceling D_n for all n at the same time, given the geometry (radius) and magnetic properties (permeabilities). However, we can cancel a particular term in the multipolar expansion of the distortion.

Indeed, we can set $D_n = 0$ (for $n > 0$) and find

$$\mu_2 = \frac{(2n+1)\mu_0}{(n+1)\left(\beta^{2n+1} - 1\right)} + \mu_0 \Longleftrightarrow \beta = \left(\frac{(2n+1)\mu_0}{(n+1)(\mu_2 - \mu_0)} + 1\right)^{\frac{1}{2n+1}}. \tag{S10}$$

Note that now the results does not depend on α. This means that for each n (for each multipolar term) and given the ratio β there is a given μ_2 that cloaks that n-term. Or, in other words, for each n and a particular μ_2 and R_2 there is a R_3 that yields cloaking of that n term. There is no way to cancel all the terms at the same time.

The case $n = 1$ is of particular interest. In this case we have

$$\mu_2 = \mu_0 \frac{2R_3^3 + R_2^3}{2(R_3^3 - R_2^3)}, \tag{S11}$$

which is exactly the same relation needed for cloaking a uniform external field (Supporting Material of Ref. [4]). In other words, for a given β, the same μ_2 that allows to cloak a uniform external field also cancels the first dipolar term of the distortion term when the bilayer is in the presence of the non-homogeneous field of the external dipole.

What about the other terms in this case? If we fix μ_2 for canceling the $n = 1$ term [Eq.(S10)], we get

$$D_n(\beta) = \frac{m}{4\pi d^2} \left(\frac{R_2}{d}\right)^n R_2^{n+1} \frac{(-1)^{n+1} n \beta^{2n+1} \left(3(n+1)\beta^{2n+1} - 4n\beta^3 + n - 2\beta^3 - 1\right)}{\left(\beta^{2n+1}\left(2(2n+1)\beta^3 - n - 2\right) - 3n\right)}. \tag{S12}$$

In particular, when the exterior shell [of permeability μ_2 given by Eq. (S11)] is very thin, that is $\beta \to 1$, the constants D_n reduce to

$$D_n(1) \simeq \frac{m}{4\pi d^2} \left(\frac{R_2}{d}\right)^n R_2^{n+1} \frac{(-1)^{n+1}(n-1)n}{4\pi(n+2)}. \tag{S13}$$

Comparing Eqs. (S12) and (S13), we see that $D_n(\beta) \geq D_n(1)$ (equality only when $\beta = 1$). That is, the thinner the exterior shell, the lower the value of each n-term.

III. OPTIMIZATION OF THE EXTERNAL METASURFACE

In the previous section we showed that a spherical FM shell surrounding an interior SC shell whose permeability fulfills Eq. (1) of the main text exactly cloaks a uniform applied field. However materials with a fine tuned intermediate relative permeability values (e.g. between 5 and 20) are difficult to find. For this reason we design an external *metasurface* consisting of several pieces of thin high-permeability FM foil (e.g. mu-metal) and we study the response given by different configurations as a function of the size and distribution of the pieces.

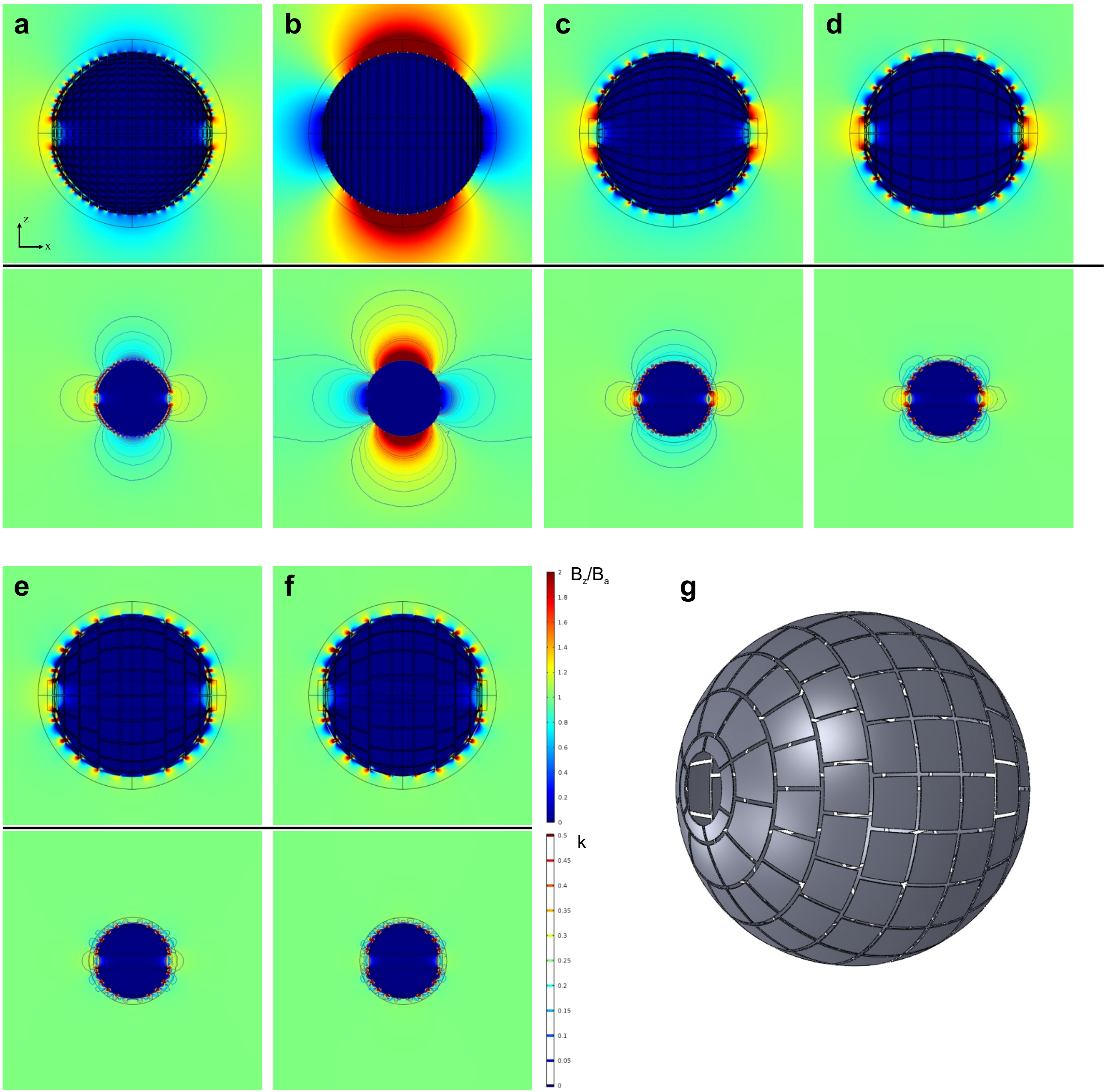

Figure S3: **a**-**f** Simulation results considering different metasurface designs surrounding an ideal superconducting shell with two traversing holes. Upper parts of the figures show plots of the field B_z/B_a along the median plane of the 3D calculations. Lower parts show distortion lines for each case, calculated as the points in the median plane where $(B_z - B_a)/B_a = k$; the better cloaking achieved, the closer the lines are to the surface. Panel **f** shows the final design, whose 3D view is shown in **g**.

In Fig. S3 we show plots of the median plane of 3D-numerical simulations considering different metasurface designs (**a**-**f**). In all the cases we consider an inner superconducting shell ($\mu^{\mathrm{SC}}/\mu_0 = 10^{-5}$) having two holes aligned with the x-axis (where the hose is going to be placed) and different configurations of ideal FM plates

($\mu^{\mathrm{FM}}/\mu_0 = 10^5$) with a thickness of 0.3mm. A uniform field perpendicular to the cylindrical hole's axis is applied $\vec{B}_a = B_a \hat{u}_z$. Upper parts of the figures show plots of B_z/B_a. Lower parts of the figures include different distortion lines, explicitly showing the distortion reduction along the optimization process. The final design is shown in Fig. S3**f** and **g**, and consists of 155 FM pieces with 6 different shapes placed at a radial distance of 43mm to the center.

Our proposal is not strictly omni-directional because the plates distribution has a preferred direction (given by the direction of the holes for the hose), but a similar optimization process could be done assuming isotropic distributions of plates wrapping the sphere (following a spherical polyhedron, for example).

IV. NUMERICAL CALCULATIONS OF THE DESIGNED WORMHOLE

The complete wormhole is simulated using full 3D finite-element calculations obtained by the AC/DC module of the Comsol Multiphysics software. In the calculations we assume the exterior FM part is a metasurface made of FM plates following the optimum design found in Section III of the Supplementary Information and the interior shell is an ideal SC shell with two holes (in which the hose is introduced). The hose is assumed to be a FM cylinder. A uniform field in the z direction B_a is applied.

The SC shell has inner and outer radii of 40 and 41mm, respectively and the traversing holes a radius of 8mm (the relative permeability of the SC is $\mu^{\rm SC} = 10^{-3}$). The FM pieces have a thickness of 0.3mm and are placed around a virtual sphere of 43mm of radius (their relative permeability is $\mu^{\rm FM} = 10^3$). The interior hose is simulated as cylinder of 12mm of diameter and a length of 86mm made of ideal FM material ($\mu^{\rm FM} = 10^3$). The coil creating the dipolar field is simulated as a cylinder of a radius 5mm and length 10mm with a uniform magnetization in the x direction (the magnetization is adjusted to create the same field as the real coil in isolated conditions). The cylinder is aligned with the hose and there is a gap of 2mm of free space between the hose and the cylinder's face.

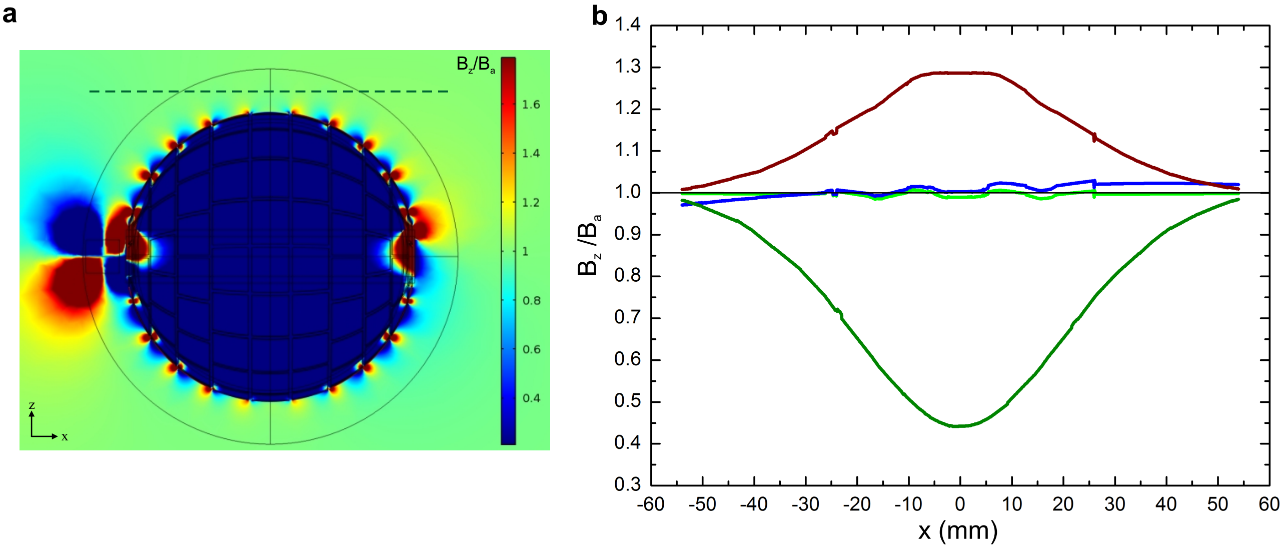

Figure S4: **a** Result of the numerical calculation, showing the line at which the field is plotted (dashed green). **b** Plots of the z-field component calculated in different situations; light green when only a uniform field is applied and blue when both the uniform and the field of the coil are applied (dark red and green are the calculated fields when only the FM exterior shell and only the inner SC shell are present, respectively, and applying only the uniform field).

From these calculations (Fig. S4) two interesting effects can be seen when the wormhole is complete. When only the uniform field is applied the distortion caused by the device is very small (light green line) and symmetric. When the small coil is turned on (blue line) the distortion slightly increases; at positions near the coil (negative x) the B_z field is decreased whilst it slightly increases at positions close to the wormhole's end. These effects are also found in the experimental measurements and can be explained by taking into account that the field lines that are transferred through the wormhole return from the wormhole's end to the coil. This field increases the z-component of the total field near the wormhole's end and decreases it near the coil, as field lines close at the coil.

V. TRANSMISSION FIELD MEASUREMENTS

The field at the end of the wormhole was measured in different situations as a function of the distance to the device's end. Before any field was applied, we measured the field at the end (dark yellow points in Fig. S5**a**); the measured field was produced by the remanent magnetization of the ferromagnetic parts. Then, the uniform field of the Helmholtz coils was applied and the field at the end was measured again (green points). Ideally, measurements should not change since the applied field is perpendicular to the component measured by the probe T, nevertheless we observed a constant increase respect to the first measurements. This shows a small component of the applied field was also measured by the probe, probably due to an imperfect alignment of the probe T respect to the coils. Finally the small coil was also fed and a clear transmitted field was measured (blue points).

From these measurements the field transmitted by the wormhole when a field is introduced at one of its ends can be calculated by subtracting the measurements when the small coil and the uniform field is applied less the measurements when only the uniform field is applied [5]. This *transmitted field*, B_{T}, corrects for the effect of the existing remanent magnetization and, therefore, only accounts for the field that is transferred from the coil to the wormhole's exit.

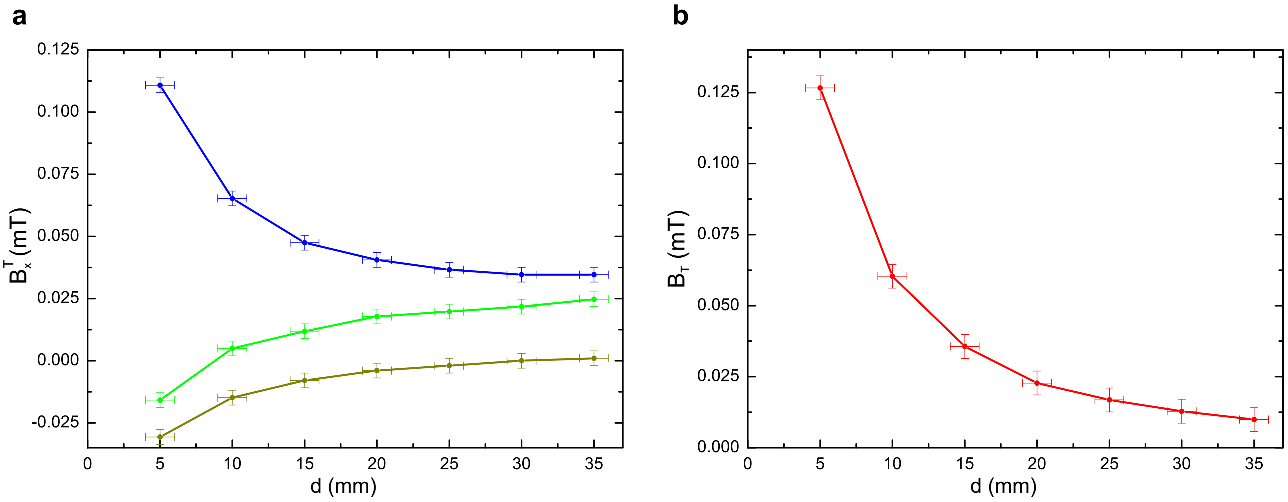

Figure S5: **a** Field measurements at the wormhole's end before any field was applied (dark-yellow points), when the uniform field was applied (green points) and when the uniform field and the field of the small coil was applied (blue points). **b** The transmitted field, B_{T}, was calculated by subtracting the values on the green line from those on the blue line in **a**.

VI. MEASUREMENTS OF FIELD DISTORTION AT DIFFERENT DISTANCES FROM THE WORMHOLE

The distortion of the uniform applied field was measured at different distances from the surface of the wormhole. In Fig. S6 we show the field measurements at 2, 5, 10 and 15mm from the surface of the device (see dashed green lines in Fig. 2B of the main text), measured with probe C. A clear systematic behavior is observed, decreasing the field distortions as we move far from the device. However notice the distortion caused by only the external FM part (dark-red lines) and only the SC shell (dark-green or dark-blue lines) are clearly measured even at a distance of 15mm from the device. At this distance, the distortion caused by the complete wormhole is effectively zero.

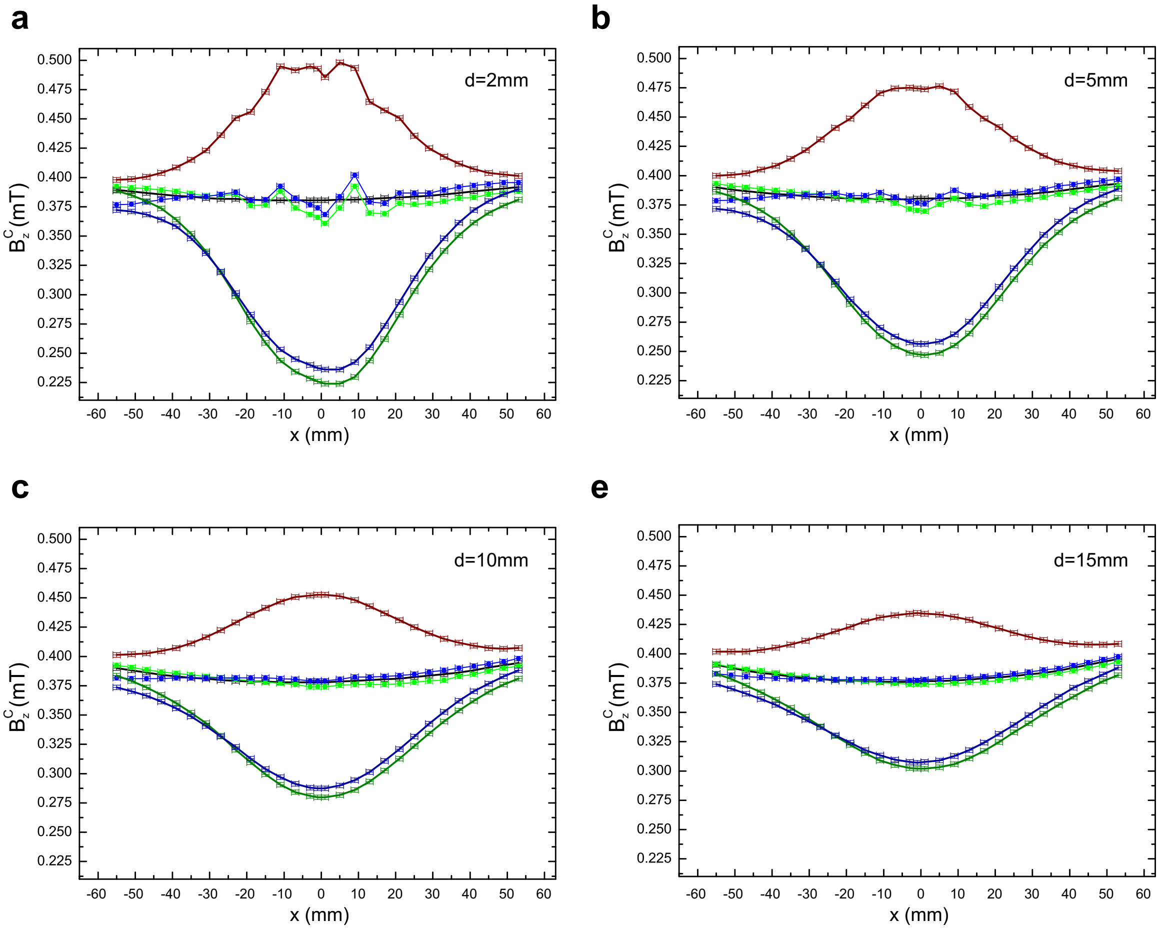

Figure S6: Field distortion measurements performed at 2, 5, 10 and 15mm from the surface (**a**, **b**, **c** and **d**, respectively). Black solid lines are measurements of the field created by the bare Helmholtz coils, dark-red lines are measurements of the wormhole when the field is applied at room temperature (i.e. the SCs are deactivated), dark-green lines are measurements performed at liquid nitrogen temperature when only the SC shell is present and the field of the Helmholtz coils is applied and dark-blue lines are measurements when also the field of the small coil is applied. Green points are measurements of the complete wormhole when only the uniform field is applied and blue points are measurements when also the field of the small coil is applied.

These plots show that when the small coil is fed the total field B_z^{C} tends to decrease at points close to the coil ($x < 0$) and to increase at points near to the wormhole's exit ($x > 0$). This effect is also observed in the numerical calculations, and is explained by the field lines transmitted through the wormhole that come out at the end and return to the coil. The contribution of these lines increase the total field B_z near the wormhole's end and decrease the total field near the coil.

[1] A. Alù and N. Engheta, Phys. Rev. Lett. **100**, 113901 (2008).
[2] A. Alù and N. Engheta, Phys. Rev. E **72**, 016623 (2005).
[3] M. Kerker, J. Opt. Soc. Am. **65**, 376 (1975).
[4] F. Gömöry, M. Solovyov, J. Souc, C. Navau, J. Prat-Camps, A. Sanchez, Science **335**, 6075 (2012).
[5] J. Prat-Camps, C. Navau and A. Sanchez, Appl. Phys. Lett. **105**, 234101 (2014).
